# Mechanisms of Immune Tolerance in Liver Transplantation-Crosstalk Between Alloreactive T Cells and Liver Cells With Therapeutic Prospects

**DOI:** 10.3389/fimmu.2019.02667

**Published:** 2019-11-19

**Authors:** Hong Lei, Petra Reinke, Hans-Dieter Volk, Yi Lv, Rongqian Wu

**Affiliations:** ^1^National Local Joint Engineering Research Center for Precision Surgery and Regenerative Medicine, The First Affiliated Hospital of Xi'an Jiaotong University, Xi'an, China; ^2^Berlin Institute of Health Center for Regenerative Therapies, Charité University Medicine Berlin, Berlin, Germany; ^3^Berlin Center of Advanced Therapies, Berlin, Germany; ^4^Institute of Medical Immunology, Charité University Medicine Berlin, Berlin, Germany

**Keywords:** liver transplantation, alloreactive T cells, crosstalk, liver cells, tolerance induction

## Abstract

Liver transplantation (LTx) is currently the most powerful treatment for end-stage liver disease. Although liver allograft is more tolerogenic compared to other solid organs, the majority of LTx recipients still require long-term immune suppression (IS) to control the undesired alloimmune responses, which can lead to severe side effects. Thus, understanding the mechanism of liver transplant tolerance and crosstalk between immune cells, especially alloreactive T cells and liver cells, can shed light on more specific tolerance induction strategies for future clinical translation. In this review, we focus on alloreactive T cell mediated immune responses and their crosstalk with liver sinusoidal endothelial cells (LSECs), hepatocytes, hepatic stellate cells (HSCs), and cholangiocytes in transplant setting. Liver cells mainly serve as antigen presenting cells (APCs) to T cells, but with low expression of co-stimulatory molecules. Crosstalk between them largely depends on the different expression of adhesion molecules and chemokine receptors. Inflammatory cytokines secreted by immune cells further elaborate this crosstalk and regulate the fate of naïve T cells differentiation within the liver graft. On the other hand, regulatory T cells (Tregs) play an essential role in inducing and keeping immune tolerance in LTx. Tregs based adoptive cell therapy provides an excellent therapeutic option for clinical transplant tolerance induction. However, many questions regarding cell therapy still need to be solved. Here we also address the current clinical trials of adoptive Tregs therapy and other tolerance induction strategies in LTx, together with future challenges for clinical translation from bench to bedside.

## Introduction

Liver transplantation (LTx) is currently the most powerful treatment for end-stage liver disease. Benefitting from advances in surgical techniques, remarkable improvements in transplant recipient survival have been achieved in the last decades since Dr. Starzl conducted the first human LTx in 1963 (1). As an immunoregulatory organ, liver allograft in the transplant setting is more tolerogenic compared to other organs such as the kidney, heart, and intestine. It is reported that almost 20% of stable and carefully selected liver transplant recipients can be weaned safely off all immunosuppression (IS) (2). However, the majority of liver transplant recipients still require open-ended, or even lifelong IS to control the unwanted alloimmune responses, which is dominantly mediated by long-term, high magnitude CD8 T cells with the help of secondary lymph nodes and CD 4 T cells. Long-term or overdose IS treatment can lead to serious side effects such as severe infections and malignancy recurrence post transplantation (3–5). Therefore, understanding the mechanism of liver transplant tolerance and crosstalk between immune cells, especially alloreactive T cells and liver cells, can shed light on more specific tolerance induction strategies for clinical translation.

The liver receives 75% of the blood from the portal vein, which is rich in antigens and microbial products originated from the stomach, gut and spleen, and 25% of the blood is oxygenated from the hepatic artery (6). Thus, the hepatic immune system is tightly controlled and regulated under physiological conditions. In addition to the leukocytes from the blood flow through the liver, the liver itself consists of hepatocytes, hepatic stellate cells (HSCs), liver sinusoidal endothelial cells (LSECs), cholangiocytes, and a diverse array of immune cells residing within or trafficking to the liver (7). Crosstalk between liver cells and immune cells plays a central role in keeping the balance of immunity and tolerance. In general, innate immune cells such as dendritic cells (DC) and liver-resident DCs (Kupffer cells) serve as professional APCs to T cells, thereby mediating hepatic immunity. Interaction of innate immune cells and liver cells has been reviewed intensively by others (8–10). As alloreactive T cells or memory T cells mediated rejection represents a major hurdle to successful transplant tolerance induction, in this review, we mainly focus on the crosstalk between alloreactive T cells and liver cells in the transplant setting together with potential therapeutic prospects for tolerance induction.

## T Cell Mediated Rejection With Alloantigen Recognition Pathways

When a liver is transplanted from the donor to the recipient, the alloantigen—mainly the allogenic major histocompatibility complex (MHC), or human leukocytes antigens (HLA) in humans—is ubiquitous, persists probably for life, and can be presented by both professional and unprofessional antigen presenting cells (APCs) at numerous sites. Thus, transplant rejection is mainly caused by the mismatch of MHCs or HLAs even in LTx. Alloantigen activated helper T cells (Th) secrete cytokines including TNFα, IFNγ, and IL-2 to further enhance the innate immune responses upon alloantigen challenge; on the other hand, they also stimulate effector CD4 T cells and cytotoxic CD8 T cells to express granzyme and perforin, thereby attacking the liver graft. In addition to the cell-mediated acute rejection, donor (graft) specific antibody (DSA) mediated humoral immune response is another important reason for hyper-acute rejection and chronic rejections. DSA mediated rejection is initiated by and in conjunction with T cell mediated alloimmunity (11–13). Several groups have shown that increased memory T cells or stem-like memory T cells correlate to allograft rejection or graft vs. host disease (GvHD) in human and animals. Stem-like memory T cells have the capacity to not only reconstitute the full diversity of memory and effector T cell population, but also maintain their own pool size through self-renewal (14–16). Therefore, memory T cells, especially donor-antigen specific memory T cells, are a major obstacle for successful tolerance induction. Moreover, as the counterpart of conventional T cells, regulatory T cells (Tregs), which are a specialized CD4 T cell subpopulation with the key transcription factor FoxP3 expression, are found to play an essential role in operational tolerance post solid organ transplantation. We showed previously that memory Tregs had superior capacity compared with naïve Tregs through higher expression of CD25 (IL-2 receptor α chain), CD39, CTLA-4 and other important molecules (17, 18). Nevertheless, the formation of immune memory initiate through alloantigen recognition and alloreactive T cells response is the backbone of adaptive immunity to allograft in the transplant setting (19). Notably, the alloimmune response is distinct from the immune response to classically pathogenic antigens because the alloreactive repertoire is highly diverse, especially in the naïve T cells subpopulation, as we showed before with next-generation sequencing (NGS) technology (18). The T cell receptor (TCR) provides a unique identity for each cell clone with around 2.5*10^7^ TCRs for human naive T cells in each individual; the TCR repertoire against a given allogenic MHC haplotype is believed to be <10% of the entire TCR repertoire (19, 20). Therefore, recognition of the alloantigen is the first critical step for the following immune response or tolerance induction in the transplant setting.

**Figure 1:**
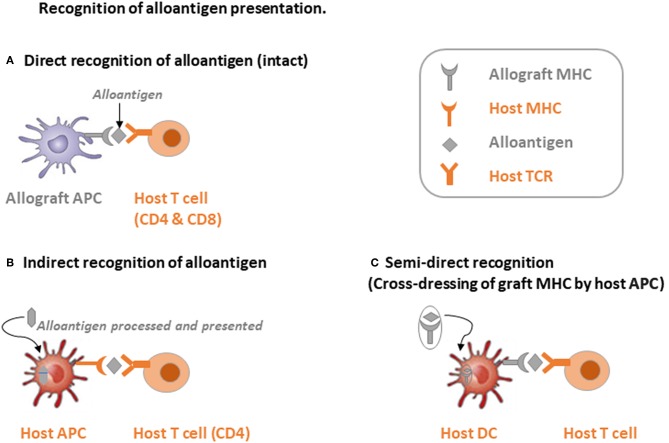
Schematic illustration of three alloantigen recognition pathways. **(A)** Direct recognition of alloantigen: allograft antigen-presenting cells (APCs) present intact alloantigen directly to host T cells. **(B)** Indirect recognition of alloantigen: host APCs process and present the allograft-derived peptides to host T cells, mainly CD4 T cells. **(C)** Semi-direct recognition way: host APCs, mainly dendritic cells (DCs), acquire graft MHC molecules, which is called cross-dressing, and present the peptide directly to host T cells.

To recognize the alloantigen by host TCRs, there are mainly 3 pathways: (i) direct way, (ii) indirect way, and (iii) semi-direct way through cross-dressing of graft MHC by host dendritic cells (DC) (19). Firstly, as shown in Figure 1A, through the direct recognition way, allograft APCs present the alloantigen with their own MHC-I molecules to the host CD8 T cells and allograft MHC-II to the host CD4 T cells. The intact antigen (protein) is recognized directly without the processing procedure. Direct recognition of the alloantigen is believed to be the dominant pathway of transplant rejection, which also includes the passenger leukocytes theory. “Passenger leukocytes” refer broadly to all the graft-derived immune cells that are transferred to the host secondary lymphoid tissue and trigger allograft rejection by direct recognition of the alloantigen (21–23). However, the contribution of passenger leukocytes to allograft rejection or tolerance induction is still not clearly understood. Irradiation of the allograft before surgery in rodent models results in killing of the graft lymphocytes and transplant rejection in otherwise tolerant recipients, suggesting the tolerance induction role of donor-derived graft-resident lymphocytes (24–26). On the other hand, the majority of donor lymphocytes are replaced by recipient bone marrow derived hematolymphoid cells within months post LTx (27–29). Nevertheless, direct recognition of the alloantigen by CD4 T cells was considered to persist at early time points after transplantation and was highly correlated with the lifespan of graft DCs (30). Whereas, indirect recognition of alloantigen is considered to be related with both acute and chronic transplant rejection. By this way, alloantigen is internalized and processed by host APCs into peptide antigens, which are further presented with host MHC molecules and thereby recognized by the TCR repertoire of host T cells (Figure 1B). CD4 T cells response from the indirect recognition way is believed to be more relevant with the allograft rejection than CD8 T cells in solid organ transplantation due to the relatively low expression of host MHC-I antigen epitopes in the vascularized allografts (31, 32). Last but not least, through the semi-direct recognition way, the host DCs acquire expression of the graft MHC molecule, which is also called cross-dressing of the host DCs, then re-present the graft MHC-antigen complex as intact alloantigen to the host T cells without further processing (Figure 1C). This phenomenon was also observed by Ono et al. (33) that in a mice LTx model, graft interstitial DCs decreased rapidly post LTx, then they were replaced by host DCs, which peaked at day 7 and persisted indefinitely. Around 60% of the host DCs in the liver graft expressed graft MHC-I, suggesting cross-dressing, and controlled the proliferation of anti-graft host T cells. On the other hand, non-cross-dressed DCs failed to suppress the anti-graft T cell response (34–38). The mechanism behind cross-dressing is believed to be related with cell-cell contact or extracellular exosomes (39–44).

Through collaboration of different alloantigen recognition pathways, host CD4 T cells are activated by continued TCR stimulation with graft MHC-II alloantigen, which are expressed either on the surface of graft APCs or re-presented by host DCs through semi-direct recognition within secondary lymphoid tissue. The principle role of the indirect pathway in CD4 T cell response, which mainly focuses on self-restricted, processed alloantigen, is likely at the late phase of transplant rejection through providing help for cytotoxic T cells and humoral immunity (45–48). The semi-direct pathway allows linked help to be delivered by indirect pathway recognition of CD4 T cells to alloreactive CD8 T cells, which target the MHC-I alloantigen expressing cells within the graft after activation and thereby exhibit cytotoxic activity through expression and secretion of granzyme and perforin (36, 42, 49). Alloantigen recognition by Tregs with different pathways, however, regulates the hepatic immune “balance” substantially more favorable for “tolerance” (50). Therefore, interaction of alloreactive T cells and APCs will be the first and key step in regulating transplant outcome in LTx.

## Crosstalk Between LSECs and Alloreactive T Cells

Within the liver allograft, there are many professional APCs such as DCs expressing low amounts of MHC antigens with co-stimulatory molecules and Kupffer cells (KCs) phagocytosing pathogens and secreting cytokines together with antigen processing and presenting (9, 51). Additionally, a large amount of non-professional APCs such as liver cells also interact with alloreactive T cells and contribute a lot to the liver transplant outcome. Composed of 50% of liver non-parenchymal cells, LSECs constitute a unique vascular bed with fenestrae organized in sieve plates without basal membrane in the liver. They interact directly with the immune cells and antigens in the blood flow, benefiting from the rich blood supply to the liver and the special liver sinusoid structure. Therefore, LSECs are also called “gatekeepers” of the hepatic immunity (52). Together with Kupffer cells, LSECs constitute the most powerful scavenger system in the body by the expression of pattern recognition receptors (PRRs) such as Toll-like receptors (TLR), scavenger receptors, and the potent endocytic capacity with their special fenestrae and loosely organized cell junctions (53, 54).

**Figure 2:**
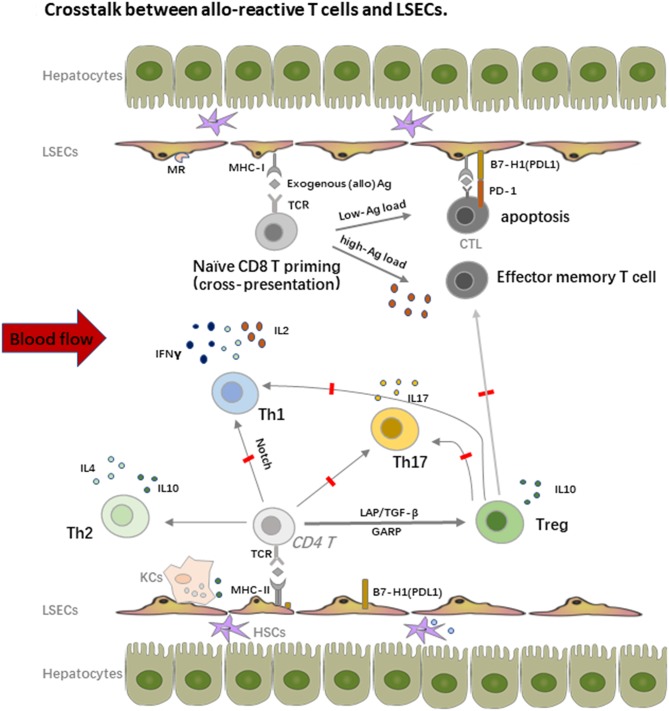
Crosstalk between alloreactive T cells and LSECs. LSECs constitute a unique vascular bed with fenestrae organized in sieve plates without basal membrane in the liver. They are the most powerful scavenger system by expression of pattern recognition receptors (PRRs), notably the mannose receptor (MR). On one hand, LSECs process and transfer the MHC-I to the naïve CD8 T cells, which is called “cross-dressing.” This priming process upregulates expression of the co-inhibitory molecule B7-H1(PDL1) on LSECs, whereas the expression of co-stimulatory molecule CD80/CD86 is not changed, thus the binding leads to the apoptosis of the alloreactive CD8 T cells. The LSECs induced tolerance is also highly correlated with antigen load and the strength of TCR stimulation. On the other hand, LSECs also prime naïve CD4 T cells with expression of MHC-II, especially under the inflammatory conditions, but fail to stimulate the proliferation of these cells due to the low expression of co-stimulatory molecules. LSECs also regulate the fate of naïve CD4 T cell differentiation within the liver graft. They suppress the differentiation of Th1 and Th17 cells but favor the enrichment of immune suppressive Th2 and Tregs, which promote the allograft tolerance.

In addition to the potent endocytosis capacity, LSECs are also the unique liver-resident APCs by expressing both MHC-I and MHC-II molecules, which take up, process and present many antigens, including alloantigens to both CD8 and CD4 T cells within the liver graft. As shown in Figure 2, LSECs can take up alloantigens through PPRs, notably the mannose receptor (MR), process and transfer them to MHC-I for the priming of naïve CD8 T cells, which is called cross-presentation as MHC-I normally exhibit endogenous antigens rather than exogenous peptides (alloantigen as foreign antigen in transplant setting) (55, 56). However, the priming of naïve CD8 T cells by LSECs upregulates the expression of the co-inhibitory molecule B7-H1(PDL1) on LSECs whereas the expression of co-stimulatory molecule CD80/CD86 is not changed, thus the binding of B7-H1 on LSECs and PD-1 on naïve CD8 T cells leads to the apoptosis of the alloreactive CD8 T cells, creating therefore the tolerogenic environment within the liver graft. Interestingly, the LSECs-induced tolerance is highly correlated with antigen load and the strength of TCR stimulation in mice. Tolerance only occurs in low-dose antigen stimulation while high-dose antigen load results in the differentiation of effector memory T cell phenotype; this process is determined partly by IL-2 secretion of naïve CD8 T cells upon early antigen priming. Furthermore, exogenous IL-2 overrides B7-H1 mediated tolerance by LSECs and induces cytotoxic T lymphocytes (CTL) differentiation (57–59). Nevertheless, LSEC lectin (LSECtin), a member of the dendritic cell-specific ICAM-3 grabbing non-integrin (DC-SIGN) family, specifically recognizes activated T cells and negatively regulates the intrahepatic immune responses (60, 61).

Similar with CD8 T cells priming, LSECs can prime naïve CD4 T cells with expression of MHC-II, especially under inflammatory conditions, but fail to stimulate the proliferation of these cells due to the low expression of co-stimulatory molecules. Importantly, LSECs also regulate the fate of naïve CD4 T cell differentiation within the liver graft. Neumann et al. found that LSECs could suppress the differentiation of pro-inflammatory Th1 cells and promote the secretion of immune suppressive cytokines such as IL10 via the Notch pathway (62). As we addressed before, Tregs are another fundamental mediator for keeping allograft tolerance. There are several different Tregs including natural Tregs (nTregs), induced Tregs (iTregs), IL10 producing Type 1 regulatory T cells (Tr1 cells), and TGF-β producing Th3 cells. nTregs are mainly developed from the thymus while iTregs are induced from naïve T cells with the presence of a low amount of antigen and TGF-β. iTregs play an essential role in keeping immune homeostasis at mucosal interfaces with expression of probably a distinct TCR repertoire as nTregs (63). Under the condition of vast antigens in the liver and TGF-β secreted by DCs, hepatic iTregs are the major source of peripheral iTregs and lead to transplant tolerance together with nTregs in both humans and mice (17, 64–68). LSECs also promote cytokine secretion of the immune suppressive Th2 cells in addition to iTregs induction in animal models (69). Furthermore, *in vitro* stimulation of Th1 and Th17 by LSECs actively inhibits their capacity to secrete IFNγ and IL17, which is tightly correlated with the dominate inhibitory (B7-H1) over co-stimulatory (CD80/CD86) signals on LSECs and IL10 production by other tolerogenic cells such as DCs (70). As Th1 and Th17 cells are important mediators of transplant rejection post LTx (71, 72), the enrichment of Tregs contributes a lot to the tolerance induction as transient accumulation of total Tregs in peripheral blood of transplant recipients, especially non-rejection recipients at 1 or 2 weeks post LTx, was observed. Similar enrichment of Tregs was also proved in tolerogenic kidney transplant recipients, suggesting the priming of T cell response by the graft antigens (17, 67, 73).

Notably, the crosstalk between LSECs and T cells largely depends on cell-cell contact by different expression of adhesion molecules and chemokine receptors. Recruitment and accumulation of CD8 T cells within the liver depend primarily on TCR activated intercellular adhesion molecule 1 (ICAM1) expressed by LSECs and slightly on vascular cell adhesion molecule 1 (VCAM1), which does not need the recognition of intrahepatic antigens, thereby passively sequestering activated CD8 T cells (74). On the other hand, liver-resident T cells express lymphocyte function-associated antigen-1 (LFA-1) (CD11a or αLβ2 integrin) rather than CD103, an integrin that is required to retrain tissue-resident T cells in many epithelial tissues, to interact with ICAM1 on LSECs (75, 76). Chemokine receptor CXCL16 with its ligand CXCR6 is also involved in intrahepatic T cell and NKT cell recruitment, whereas Tregs bind to different chemokines due to their expression of CCR5 or CCR4; they are also reported to use distinct combination of adhesion receptors such as stabilin 1 to migrate cross LSECs (77).

## Interactions of Hepatocytes and Alloreactive T Cells

**Figure 3:**
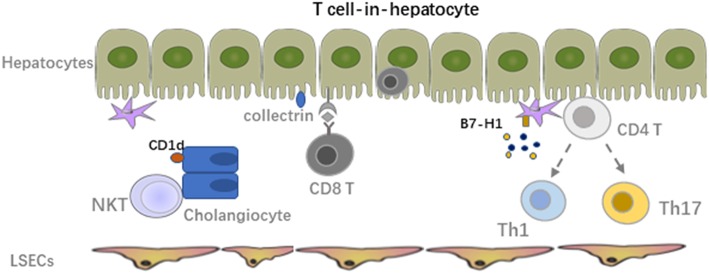
Crosstalk between alloreactive T cells and hepatocyte, HSCs, cholangiocytes. The survived lymphocytes from LSECs immune surveillance can transmigrate across the LSECs line to crosstalk with hepatocytes. The paracrine factors secreted by hepatocytes also accelerate the recruitment of lymphocytes. Hepatocytes mainly serve as non-professional APCs with expression of MHC-I to interact with CD8 T cells under physiological conditions while expression of MHC-II is also inducible under inflammatory condition especially in the presence of IFNγ. Interaction of HSCs with activated alloreactive T cells mainly lead to apoptosis of these T cells due to the low MHC-I expression. Expression of MHC-I like molecule CD1d on cholangiocytes can activate NKT cells and mediate inflammation in the bile ducts.

Through interaction of immune cells with LSECs and adhesion cascade in the hepatic sinusoids, the survived lymphocytes from the LSECs immune surveillance can transmigrate across the LSECs line with help from the orchestra of chemokines and adhesion molecules through several different routes paracellularly, transcellularly, or intracellularly, to finally get a chance to crosstalk with hepatocytes (52). The paracrine factors that were secreted by hepatocytes also accelerate the recruitment of lymphocytes. The interaction of hepatocytes and immune cells plays an important role in inducing liver transplant tolerance. In general, hepatocytes mainly serve as non-professional APCs with expression of MHC-I to interact with CD8 T cells under physiological conditions while expression of MHC-II is also inducible under inflammatory conditions, especially in the presence of IFNγ. However, low expression of co-stimulatory molecules on hepatocytes leads to apoptosis of the alloreactive T cells (10). Paul-Heng et al. have found that direct recognition of hepatocyte expressed MHC-I alloantigen (cross presentation) is required for tolerance induction, whereas the indirect recognition of the processed and presented allogeneic peptide on MHC-II by CD4 T cells is not sufficient for tolerance induction although it can prolong the graft survival and generate Tregs to promote transplant tolerance (78, 79). Additionally, processing of the soluble antigens into peptide presented by MHC-I is impaired in hepatocytes lacking collectrin, which is an intracellular chaperone protein within the endoplasmic reticulum-Golgi intermediate compartment and positively regulated (80). Different from other liver cells, hepatocytes can produce exosomes to control the active T cells response and clear the activated T cells through the non-apoptotic way of suicidal emperipolesis (SE), which is a process leading to cell-in-cell structures and promotes cell death through degradation within endosomal/lysosomal compartments (Figure 3) (81, 82). Recently, Beringer et al. have found that the interaction of hepatoma HepaRG and human peripheral blood mononuclear cells (PBMCs) in the inflammatory response can be divided into two phases. At the early phase, PBMC-HepaRG interaction can modulate the T cell polarization into Th1 cells and suppress the differentiation into Th17 cells through direct cell-cell contact with increased secretion of IL6, IL8, CCL20, and MCP-1 (Figure 3), whereas the PBMC-hepatocyte crosstalk at the late phase may down-regulate the immune response with decreased expression of HLA-DR on hepatocytes to induce the immune tolerance in the liver (83). Moreover, it is not clear yet whether the similar kinetic interaction of alloreactive T cells and hepatocytes also exist in the LTx setting.

## Interactions of HSCs, Cholangiocytes, and Immune Cells

Hepatic stellate cells (HSCs), also known as perisinusoidal cells or fat-storing cells, are crucial in liver inflammation and fibrosis by producing inflammatory and fibrotic mediators. In the context of LTx, migrating host immune cells also interact with graft liver resident cells. Both cell-cell contact and soluble cytokines or factors contribute to the graft function and transplant outcome. Inflammatory cell derived IL17A induced HSC to express collagen I directly and TGF-β from activated KCs induced expression of collagen I on HSCs indirectly, promoting the graft fibrosis progression (84). Activated HSCs produce inflammatory cytokines and chemotactic factors to accelerate the migration and deposition of immune cells, which could be further enhanced by paracrine signals from damaged hepatocytes (85–87). However, due to the low amount of MHC-I expression and co-inhibitory molecule B7-H1 on HSCs, interaction of HSCs with activated alloreactive T cells mainly leads to apoptosis of these T cell. In addition, mature HSCs can stimulate allogeneic Treg proliferation with the manner of cell-cell contact and enhance the suppressive capacity of Tregs regarding inhibiting of Teff proliferation *in vitro*. Adoptive transfer of HSC-stimulated Tregs significantly reduced liver injury in mice with autoimmune hepatitis by modulating the balance between Tregs and Th17 cell responses (88).

Cholangiocytes express MHC-I under physiological conditions and a low amount of MHC-II only in the context of inflammation (89). It was reported that through expression of MHC-I like molecule CD1d, murine cholangiocytes could present both exogenous (cross-presentation) and endogenous lipid antigens to NKT cells and activate them to mediate inflammation in the bile ducts. The human cholangiocytes also present exogenous antigens in a CD1d-restricted way to invariant NKT cells. However, CD1d expression was down-regulated in the biliary epithelium of patients with late primary sclerosing cholangitis and primary biliary cirrhosis compared to healthy controls, suggesting their potential role in the pathology of these diseases (90, 91). On biliary epithelial cells (BECs) in biliary atresia patients, increased ICAM-1 expression was also observed in association with MHC-I, but not MHC-II. The major lymphocytes within the portal tracts are CD4 T cells expressing LFA-1, indicating the potential crosstalk between them (92). MHC-I expression level on cholangiocytes might correlate with cholangitis post LTx. Interestingly, BECs express a relatively higher amount of MHC-I compared with other liver cells (12).

## Therapeutic Targets for Liver Transplant Tolerance Induction

Operational tolerance, characterized with stable graft function in the absence of IS for at least 1 year, is the final goal of all allogenic solid organ transplantation (SOT). To achieve this, several approaches for immune modulation, including adoptive cell therapy, have been conducted in the clinical trials. We and others have showed that both recipient and donor Tregs play an essential role in maintaining the graft tolerance in SOT (17, 18, 93–95). Adoptive Treg-based therapy is a very promising approach to support allograft acceptance with minimizing or potentially eliminating IS treatment. A phase II international multicenter proof-of-concept clinical trial of Treg therapy for SOT patients has been conducted in the European Union (The ONE Study). Our group have shown that nTregs from even end-stage renal disease patients could be expanded *ex-vivo* for adoptive cell therapy, whereas alloantigen specific Tregs exhibit superior immune suppressive capacity for tolerance induction (96, 97). Moreover, adoptive Treg transfer in the inflammatory phase of viral-induced myocarditis protects the heart against inflammatory damage and fibrosis via modulation of monocyte differentiation in favor of the anti-inflammatory Ly6C^low^CCR2^low^Cx3Cr1^high^ subset (98). In the liver transplant setting, Todo et al. have published a very exciting pilot study that 7 of the 10 liver transplant recipients receiving a single dose of donor antigen specific Tregs and splenectomy become operationally tolerant (99). Several clinical trials for adoptive cell therapy employing either *ex vivo* expanded polyclonal Tregs or alloantigen specific Tregs are also being conducted worldwide. The ThRIL trial at King's College Hospital, UK [clinical trials.gov NCT02166177] utilizes polyclonal Tregs in their therapeutic setting. The DeLTA and ARTEMIS trials at University of California, San Francisco, USA, use donor antigen reactive Tregs for tolerance induction in both deceased donor LTx [NCT02188719] and living donor LTx [NCT02474199]. These clinical trials will not only show the efficiency and safety of Treg therapy but also indicate the survival and homing of these adoptively transferred cells as they are labeled with deuterium (100). Another clinical trial at Nanjing Medical University, China, utilizes donor antigen specific Tregs for chronic rejections in LTx patients at early and late time points, with multiple Treg injections and IS withdrawal [NCT01624077] (101).

In addition to *ex vivo* expansion of Tregs for adoptive cell therapy, other strategies regarding *in vivo* expansion of Tregs are also very appealing. For instance, low-dose IL-2 administration could expand Tregs *in vivo* up to 8 times without a significant increase in Teff cells because Tregs express a higher amount of IL-2 receptor α-chain (CD25) and thus respond to a very low amount of IL-2 while Teff could not. This brings the possibility to expand Tregs pool *in vivo* without requirement of very expensive and large-scale GMP facilities for clinical grade Treg products (102). Low dose IL-2 also restores Treg homeostasis or dysfunction in chronic GvHD patients (103, 104). A corresponding phase IV clinical trial, LITE Trial (NCT02949492), is in progress at King's College London. Scientists there are using low dose IL-2 to promote the selective expansion of endogenous Tregs in liver transplant recipients at the time of immunosuppression (101). Recently, Ratnasothy et al. even showed that IL-2 treatment in mice preferentially enhances the proliferation of the adoptively transferred allospecific Tregs in an antigen-dependent manner and increases the expression of regulatory-related markers, such as CTLA4 and inducible co-stimulator (ICOS). Based on this, combination therapy of both low-dose IL-2 and adoptively transferred alloantigen specific Tregs could provide an appropriate condition to enhance the immunoregulation toward alloimmune response in clinical transplantation (105). Low-dose IL-2 enriched Treg therapy is also investigated intensively in autoimmune diseases and GvHD after hematopoietic stem cell transplantation (106–109).

Notably, as antigen specific Tregs are superior to polyclonal Tregs in controlling Teff responses, improving approximately 100-fold of the efficacy, and theoretically safer due to avoiding bystander compromised immunity (110, 111), it is more appealing to use this Treg population for adoptive Tregs therapy. However, expansion of these Tregs *in vitro* is a big obstacle for clinical translation. Therefore, engineering human T cells to express a chimeric antigen receptor (CAR) is a new approach to create antigen specific T cells. For instance, autoantigen-based chimeric immunoreceptors can direct T cells to kill autoreactive B lymphocytes through the specificity of the B cell receptor (BCR) (112). Meanwhile, CAR Tregs can also be generated with CAR technology to develop alloantigen specific Tregs, which have showed potent and markedly enhanced therapeutic potential for the protection of allografts (113–115). Co-administration of antigen with tolerogenic nanoparticles (tNPs), which comprised of biodegradable polymers with encapsulated rapamycin, could inhibit ag-specific transgenic Teff proliferation and induce ag-specific Tregs. This suggests another potential strategy to expand ag-specific Tregs *in vivo* and suppress T cell-mediated autoimmunity or graft rejection (116–118). On the contrary, Treg plasticity refers to their capacity to produce inflammatory cytokines and lose FoxP3 expression (119, 120). In this case, they could transform into pathogenic Teff cells, thus contributing to disease pathogenesis, which might represent a risk for adoptive Tregs therapy. Based on these concerns, genetic “editing” through CRISPR-associated protein 9 (Cas9) system could generate optimal Tregs while ensuring stability (121, 122). However, our group have recently published that gene-editing with CRISPR-Cas9 system might cause significant safety issues because of the pre-existing ubiquitous effector T cell response directed toward the Streptococcus pyogenes (SpCas9) within healthy humans. Therefore, modification of Tregs with the CRISPR-Cas9 system still needs further careful evaluation (123).

Similar to Tregs, regulatory B cells (Bregs) function as a form of active immune regulation, which was first reported experimentally through anti-CD45RB treatment of mice receiving a cardiac allograft (124). Moreover, the field of Breg-mediated tolerance is relatively immature and their function is somehow also related with Tregs (125, 126). Regulatory DCs (DCregs) with capacity to suppress allograft rejection and promote transplant tolerance in pre-clinical models can readily be generated from bone marrow precursors or circulating blood monocytes. Donor-derived DCregs are short-lived but can induce robust donor-specific T cell hyporesponsiveness. Infusion of donor-derived DCregs could achieve IS withdrawal in patients 18 months post LTx (38, 127). Furthermore, down-regulation of HLA-1 expression level on hepatocytes can reduce the strength of allogeneic immune responses and improve the graft survival. Alternatively, gene transfer of alloantigen to hepatocytes induces the expansion of CD8 Tregs, which further prevent the allograft rejection in mice pancreatic islets transplantation. These gene-modified hepatocytes may also provide some possible tolerance induction strategy in the future (128, 129).

## Summary and Outlook

Based on the alloimmune responses mediated transplant rejection, interactions of alloreactive T cells with both innate immune cells and liver cells including hepatocytes, LSECs, HSCs, and cholangiocytes contribute dramatically to the transplant outcome. The capacity of alloantigen presenting and inflammatory mediator secretion by liver cells dominates the fate of alloreactive T cell differentiation and transplant outcome. As Tregs play an essential role in inducing and maintaining the allograft tolerance, Treg based therapy either with adoptively transferred *ex vivo* expanded Tregs or low-dose IL-2 *in vivo* enriched Tregs pool is very promising and appealing for clinical translation. However, more efficient Treg expansion protocols have to be developed and evaluated to improve the efficiency of the therapy and reduce the cost for the clinical cell products. In addition, combination of several tolerance induction strategies might provide synergistic results, but more clinical studies from multiple centers still need to be conducted for successful translation from bench to bedside.

## Author Contributions

All authors listed have made a substantial, direct and intellectual contribution to the work, and approved it for publication.

### Conflict of Interest

The authors declare that the research was conducted in the absence of any commercial or financial relationships that could be construed as a potential conflict of interest.
